# Supplementary material for: Neutron star mass estimates from gamma-ray eclipses in spider millisecond pulsar binaries
Source: Nat Astron. 2023 Jan 26;7(4):451–62. doi: 10.1038/s41550-022-01874-x (PMC10119022; doi:10.1038/s41550-022-01874-x)
Supplement: Supplementary file 1 — Supplementary Tables 1 and 2. [file 41550_2022_1874_MOESM1_ESM.pdf]

nature astronomy

Article

https://doi.org/10.1038/s41550-022-01874-x

Neutron star mass estimates from gamma-ray eclipses in spider millisecond pulsar binaries

In the format provided by the
authors and unedited

Supplementary Table 1: Results of eclipse searches from candidate pulsars. For the two systems with false-alarm probabilities $P_{\rm FA} < 1\%$ we give the minimum and maximum eclipse durations for the orbital parameters with the maximum eclipse likelihood, and the inclination $i^{\rm min}$ at which the minimum eclipse duration is reached for a Roche-lobe filling companion star, with assumed mass ratio $q = 5$. For 4FGL J2333.1−5527, the eclipse durations are for the more significant detection at the revised value of $T_{\rm asc}$ found outside the initial search range (see text).

Source	Class	$\delta \log \mathcal{L}_{\rm max}$	$P_{\rm FA}$	$\theta_{\rm min}$	$\theta_{\rm max}$	$K_{\rm c}$ (km s^{-1})	$i^{\rm min}$ (°)	Ref.
4FGL J0212.1+5321	RB	8.22	0.4			214.1 ± 5.0		[1, 2]
4FGL J0336.0+7502	BW	4.74	0.7			—		[3]
4FGL J0523.3−2527	RB	4.29	0.9			190.3 ± 1.1		[4]
PSR J0838−2827	RB	10.82	0.003	0.015	0.023	315.0 ± 17.0	75.80	[5]
4FGL J0940.3−7610	RB	7.19	0.5			293.2 ± 6.0		[6]
PSR J0955−3949	RB	6.34	0.5			272.0 ± 4.0		[7]
PSR J2333−5526	RB	11.31	0.001	0.066	0.079	360.0 ± 5.0	81.09	[8]

Supplementary Table 2: Constraints for pulsars without detected eclipses, and with no published companion radial velocity amplitudes. Inclination upper limits are derived assuming $q = 70$, and a 50% Roche-lobe filling companion.

Pulsar	Class	$\delta \log \mathcal{L}$	$\theta^{\rm max}$	$i^{\rm max}$ (°)
J0023+0923	BW	0.08	0.007	85.9
J0251+2606	BW	6.03	0.031	90.0
J0312−0921	BW	2.28	0.007	86.0
J0610−2100	BW	0.08	0.003	85.8
J0636+5129	BW	0.67	0.062	90.0
J1124−3653	BW	2.02	0.012	86.3
J1446−4701	BW	0.10	0.004	85.8
J1513−2550	BW	0.00	0.016	86.9
J1544+4937	BW	0.07	0.007	85.9
J1641+8049	BW	0.09	0.007	85.9
J1745+1017	BW	1.09	0.007	86.0
J1805+0615	BW	0.30	0.013	86.5
J1833−3840	BW	0.45	0.044	90.0
J1908+2105	BW	0.98	0.015	86.8
J1946−5403	BW	1.02	0.005	85.8
J2017−1614	BW	1.13	0.015	86.7
J2047+1053	BW	0.98	0.011	86.2
J2051−0827	BW	0.29	0.015	86.7
J2052+1218	BW	0.96	0.040	90.0
J2115+5448	BW	2.24	0.010	86.2
J2214+3000	BW	0.00	0.000	85.8
J2234+0944	BW	1.21	0.006	85.9
J2241−5236	BW	0.00	0.001	85.8
J2256−1024	BW	2.21	0.010	86.1

References

[1] Li, K.L. *et al.*, Discovery of a Redback Millisecond Pulsar Candidate: 3FGL J0212.1+5320. *Astrophys. J.* **833**, 143 (2016).

[2] Linares, M. *et al.*, A millisecond pulsar candidate in a 21-h orbit: 3FGL J0212.1+5320. *Mon. Not. R. Astron. Soc.* **465**, 4602–4610 (2017).

[3] Li, K.L., Jane Yap, Y.X., Hui, C.Y. & Kong, A.K.H., Revealing a New Black Widow Binary 4FGL J0336.0+7502. *Astrophys. J.* **911**, 92 (2021).

[4] Strader, J. *et al.*, 1FGL J0523.5-2529: A New Probable Gamma-Ray Pulsar Binary. *Astrophys. J. Lett.* **788**, L27 (2014).

[5] Halpern, J.P., Strader, J. & Li, M., A Likely Redback Millisecond Pulsar Counterpart of 3FGL J0838.8-2829. *Astrophys. J.* **844**, 150 (2017).

[6] Swihart, S.J. *et al.*, Discovery of a New Redback Millisecond Pulsar Candidate: 4FGL J0940.3-7610. *Astrophys. J.* **909**, 185 (2021).

[7] Li, K.L. *et al.*, Multiwavelength Observations of a New Redback Millisecond Pulsar Candidate: 3FGL J0954.8-3948. *Astrophys. J.* **863**, 194 (2018).

[8] Swihart, S.J. *et al.*, A New Likely Redback Millisecond Pulsar Binary with a Massive Neutron Star: 4FGL J2333.1-5527. *Astrophys. J.* **892**, 21 (2020).
